# Anatomical Correlates of Cognitive Functions in Early Parkinson's Disease Patients

**DOI:** 10.1371/journal.pone.0064222

**Published:** 2013-05-22

**Authors:** Roberta Biundo, Massimiliano Calabrese, Luca Weis, Silvia Facchini, Gianluigi Ricchieri, Paolo Gallo, Angelo Antonini

**Affiliations:** 1 Department for Parkinson's Disease, “Fondazione Ospedale San Camillo,” I.R.C.C.S., Venice, Italy; 2 La Clinica Neurologica, A.O. Universitaria, Padova, Italy; 3 Department of Neurosciences SNPSRR, Neurology Clinic St Anthony Hospital, University of Padua, Padua, Italy; Centre Hospitalier Universitaire Vaudois Lausanne - CHUV, UNIL, Switzerland

## Abstract

**Background:**

Cognitive deficits may occur early in Parkinson's disease (PD) but the extent of cortical involvement associated with cognitive dysfunction needs additional investigations. The aim of our study is to identify the anatomical pattern of cortical thickness alterations in patients with early stage PD and its relationship with cognitive disability.

**Methods:**

We recruited 29 PD patients and 21 healthy controls. All PD patients performed an extensive neuropsychological examination and 14 were diagnosed with mild cognitive impairment (PD-MCI). Surface-based cortical thickness analysis was applied to investigate the topographical distribution of cortical and subcortical alterations in early PD compared with controls and to assess the relationship between cognition and regional cortical changes in PD-MCI.

**Results:**

Overall PD patients showed focal cortical (occipital-parietal areas, orbito-frontal and olfactory areas) and subcortical thinning when compared with controls. PD-MCI showed a wide spectrum of cognitive deficits and related significant regional thickening in the right parietal-frontal as well as in the left temporal-occipital areas.

**Conclusion:**

Our results confirm the presence of changes in grey matter thickness at relatively early PD stage and support previous studies showing thinning and atrophy in the neocortex and subcortical regions. Relative cortical thickening in PD-MCI may instead express compensatory neuroplasticity. Brain reserve mechanisms might first modulate cognitive decline during the initial stages of PD.

## Introduction

The contribution of frontostriatal dysfunction to cognitive abnormalities in Parkinson's disease (PD) has been extensively investigated [1]. Recently, it has emerged that PD patients show a wide and variable spectrum of cognitive deficits involving multiple domains like memory, visuo-spatial and less frequently language, which are underlined by variable neuroanatomical substrates [2]–[8]. Imaging studies, using voxel-based morphometry (VBM) revealed regional brain atrophy in PD (with and without cognitive deficits) compared with healthy control. In particular visuo-spatial and visuo-perceptive impairment was associated with atrophy in temporo-parietal regions [4] while memory deficits were linked with hippocampal atrophy in patients with visual hallucinations and dementia [9]. Others studies found frontal-temporal and parietal cortex atrophy also in non-demented PD [10]–[13]. However, VBM may be insufficient to detect early anatomical changes mainly because of technical limits such as inadequate definition of grey matter intensity, surface area and cortical folding patters. The recent introduction of the surface-based cortical thickness analysis may partially minimize these problems [14] and provide complementary information to VBM in exploring the topography of brain cortical changes. Only a few studies are available in PD and each has significant limitations particularly in respect to the extent of neuropsychological assessment.

In our study we measured cortical thickness with brain MRI and used an extensive cognitive assessment in a cohort of PD patients and healthy controls. We aimed at identifying regional structural changes that best characterize PD and at investigating the relationship between regional cortical alterations and cognitive abnormalities.

## Methods

### Participants

We recruited all participants from a database build up between December 2010 and December 2011 at the Parkinson's disease Unit of the ‘San Camillo’ Hospital (Venice Lido, Italy) and at the Neurology Clinic of the University Hospital of Padua, Italy. We included all non-demented PD patients according to published diagnostic criteria for PD and PD-dementia [15]–[16] who underwent an extensive neuropsychological battery and had the same MRI scan protocol (3D sequences made with the same machine). We only considered PD patients who were on stable medications and did not report motor fluctuations. We did not include subjects with significant cardiovascular problems and history of major psychiatric disorders (i.e. bipolar disorder or major depression). Further, we excluded patients with atypical Parkinsonism and PD who had neurosurgical procedures (including deep brain stimulation). Finally we discarded scans from individuals who had movement artefacts and significant white matter hypo-intensity. Our population consisted of total 29 patients and 21 healthy controls remained. L-dopa equivalent daily dose (LEDD) and/or dopamine agonist daily dose (DAED) for each patient was calculated as previously published [17]. We collected demographic data (age, gender and education level) and clinical details (age at onset and disease duration). All PD patients had asymmetric symptoms onset (17 in the right and 10 in the left body side) except for 2 patients with bilateral symptoms at the onset. The severity of extrapyramidal symptoms was graded using the Hoehn and Yahr (H&Y). The mean H&Y score was 1.6 (SD 0.6) (12 patients had a score of 1, 3 had a score of 1.5, 11 had a score of 2, 1 had a score of 2.5 and 2 had a score of 3). Written informed consent was obtained from all participants after the nature of the study was fully explained. The study was approved by the ethic committee of the IRCCS San Camillo, Venice, Italy.

### Neuropsychological examination

Neuropsychological evaluation included the Mini Mental State Examination (MMSE) [18] for general cognitive functions, the Frontal Assessment Battery (FAB) for frontal functioning [19], the Category and Letter fluency tasks to evaluate response generation and maintenance of task instruction in working memory [20]–[21], tests of short and long term memory (RVLT, Digit Span Forward and Backwards and ROCF immediate recall) verbal and non verbal [22]–[23], tests of visual-spatial planning and attention such as the Rey-Osterrieth Complex Figure Test (ROCF) (copy and immediate recall) and Digit Cancellation [22]–[23], the Stroop Color/Word Interference Test to evaluate response monitoring and conflict resolution [24], the Trail Making Test part A to assess visual scanning, numeric sequencing and visual-motor speed (TMT-A), part B to assess general frontal lobe dysfunction (TMT-B) and the time differences between TMT-A and TMT-B (TMTB-A) to evaluate shifting abilities [25], clock drawing test (CDT) to evaluate semantic knowledge, planning and visual-spatial abilities [26] and drawing coping test (to assess visual-constructive abilities and constructional-apraxia respectively [22]. We used the Beck Depression scale (BDI-II) to assess presence of depression (range score 0–63) and measure severity of depressive symptoms. In addition all patients were interviewed for presence of hallucinations. Standardized, published normative datasets were used as comparative references to determine impairments. Tests were administered at the time of the scanning.

### Mild Cognitive Impairment definition

Diagnosis of mild cognitive impairment (MCI) was based on Movement Disorder Society task force recommendation criteria [27]. The following cognitive domains were considered: 1) attention/working memory abilities 2) executive abilities 3) visual-spatial abilities 4) memory and 5) language abilities. For each domain at least two tests were included. Its definition was based on performance in the five cognitive domains. To this end, raw individual test values were converted into Z-scores using relative Italian published normative data, corrected for age and education when possible. Patients performing ≤1.5 SD Z-score below the population mean in at least two tests for the same cognitive domain or one test in at least two different domains were classified as PD with mild cognitive impairment (PD-MCI).

### MRI acquisition and imagine processing

All images were acquired using a 1.5 T MR machine (Achieva, Philips Medical Systems, Best, The Netherlands) with 33 mT/m power gradient and a 16 channels head coil. No major hardware upgrades of the scanner occurred during the study period, and bimonthly quality assurance sessions took place to guarantee measurement stability. The following images were acquired from each subject: 1) *Fast fluid attenuated inversion recovery (FLAIR):* repetition time (TR) = 10000 msec, echo time (TE) = 120 msec, inversion time (TI) = 2500 msec, echo train length (ETL) = 23, 50 contiguous axial slices with a thickness = 3.0 mm, a matrix size = 172×288, and a field of view (FOV) = 250×200 mm. 2) Three *3D fast field echo (FFE) sequence:* 120 contiguous axial slices with TR = 25 msec, TE = 4.6 msec, flip angle = 30°, slice thickness = 1.2 mm, matrix = 256×256 and a FOV = 250×250 mm. All images were carefully inspected avoiding the presence of motion artefacts to ensure accurate identification of GM/WM boundary and the pial surface.

**Cortical thickness automatic parcellation:** The cortical thickness parcellation was carried out using Freesurfer toolkit (http://surfer.nmr.mgh.harvard.edu/) [28] with a surface–based approach performing the reconstruction of the brain surface from structural data [29]. The detailed procedure for the surface construction has been described and validated in previous publications [29]–[31]. Briefly the processing involved intensity normalisation, skull stripping, segmentation of white matter, tessellation of the grey/white matter boundary and automatic topology correction. This surface was then used as the starting point for a deformable surface algorithm to find the grey/white and grey/cerebrospinal fluid (CSF) surfaces. This method used both intensity and continuity information from the surfaces in the deformation procedures to produce representations of cortical thickness, calculated as the average of the closest distance from the grey/white boundary to the grey/CSF boundary and the grey/CSF boundary to the grey/white boundary at each vertex on the tessellated surface After registration to a common surface template resulting from the surface-based averaging technique. The cortical thickness values were extracted from 74 labelled regions per hemisphere [32]. The automated anatomical labelling volume of interests (VOIs) corresponding to the Desikan atlas regions as defined in Destrieux et al. [33] were created in the MNI standard space using the WFU-pickatlas 3.04 SPM8 tool. The 50 maps of cortical thickness were then created by an automated process obtained with a FSL 4.9 pipeline multiplying every Automated Anatomical Labelling (AAL) VOI by the corresponding cortical thickness value [modified method from Li G. et al. [34]].

**Cortical thickness group analysis:** A second level unpaired t-test group analysis was performed using a region by region voxel-based approach implemented in SPM8, masking for MNI standard GM Template. Four contrasts were analysed: PD vs. healthy controls (HC), PD without cognitive impairment (PD-CNT) vs. HC, PD-MCI vs. HC and PD-MCI vs. PD-CNT. Age and disease duration were included as covariates. Uncorrected p-value threshold of 0.05 was used. For each comparison the significant Cortical Thickness change was identified using Monte Carlo simulation in AlphaSim tool of Analysis of Functional NeuroImages (AFNI) software [35] at the individual voxel level of |t|>2.0 (df = 25, p<0.05) with a minimal cluster size of 716 contiguous voxels (3376 µl). This yielded a corrected alpha of p<0.05. The obtained statistical 3D maps were then overlaid onto the MNI standard template by using the SPM8 visualization tool. Areas of significant difference that did not survive after correction are reported as significant trend.

**Analysis of subcortical volumes:** To analyse subcortical volumes obtained from Freesurfer anatomical parcellation we applied a Bonferroni correction.

**Statistical Analysis:** Clinical and demographic variables were analysed using Pearson Chi-square test to assess differences in the distribution of dichotomous variables. T-test and Kruskall-Wallis test was applied in order to assess differences for the continuous variables.

Pearson' r bivariate correlation analysis was run between areas of cortical alterations in PD-MCI and neuropsychological tests (z scores). All statistical analyses were performed using SPSS 20.0.

## Results

### Clinical

The whole PD group had a mean age of 60.6 years (SD 10.2), mean education of 10.44 (SD 4.6), mean disease duration of 5.6 (SD 5.2) and mean age of onset of 55.4 (SD 9.8). HC had a mean age of 54.8 (SD 4.7) and education of 10.7 (SD 4.1). No differences between PD and HC in gender (p = 0.15) and education (p = 0.8) were observed. However, age was significant different (p<0.05) and was used as covariate in all groups contrast. There were 14/29 PD-MCI patients. PD subgroups analysis showed that PD-CNT and PD-MCI did not differ for demographic or clinical variables (see table 1 for details).

**Table 1:** Demographic and clinical characteristics of PD subgroups.

	PD-CNT (n = 15)	PD-MCI (n = 14)	P values
**Gender (m/f)**	10/5	7/7	0.38
**Age (y)**	59.1(11)	62.1(9.5)	0.44
**Education (y)**	11.3(5.3)	9.5(3.7)	0.38
**Disease Duration (y)**	5.8(5.3)	5.5(5.2)	0.91
**Age of onset (y)**	54.4(10.9)	56.4(8.6)	0.81
**H&Y stage**	1.6(0.7)	1.7(0.6)	0.59
**LEDD**	538.88(357.4)	652.55(342.9)	0.19
**DAED**	126.7(85.1)	139.35(82.2)	0.84
**BDI-II**	6.3(5.7)	9.8(6.8)	0.09

### Cortical thickness analysis

**Whole PD group vs. HC:** There was a significant trend for PD cortical thinning in the following areas: in the right hemisphere, in the occipital superior area, in the cuneus and precuneus, in the superior orbito-frontal area, in the olfactory area and angular gyrus. In addition we found PD volume reductions in the putamen and nucleus accumbens bilaterally and in the right caudate, hippocampus, amygdale and pallidum. The right cuneus, hippocampus, amygdale and accumbens area were significant also after correction. No significant decrements were found in the HC when compared with PD group.

**PD-CNT vs. HC:** PD-CNT showed a significant trend for cortical thinning in the right hemisphere in the middle and superior orbito-frontal area, rectus, olfactory, fusiform and precuneus areas, paracentral lobule and cuneus area, superior occipital area, supramarginal area, as well as inferior parietal and angular area. In the left side we found cortical thinning in the occipital inferior area. All these areas with the exception of the supramarginal gyrus, rectus, fusiform, and angular areas remained significant after correction. No areas of cortical thinning were found in the HC.

**PD-MCI vs. HC:** The following areas showed a significant trend of PD-MCI cortical thinning: cuneus, angular, fusiform and olfactory area, superior occipital area, superior temporal pole, middle orbito-frontal and supramarginal area in the right hemisphere. The cuneus and olfactory areas remained significant also after correction. No significant alterations were found in the HC.

**PD-MCI vs. PD-CNT:** Significant cortical thickening in PD-MCI was observed in the following regions: the superior and inferior parietal areas, precuneus, paracentral lobule, superior frontal areas and superior orbito-frontal area in the right side; the superior, middle and inferior temporal areas, the middle occipital area in the in the left side. All areas with the exception of the superior and middle temporal, and superior parietal areas were significant also after correction. There were no significant cortical thickness changes in PD-CNT.

**Figure 1:**
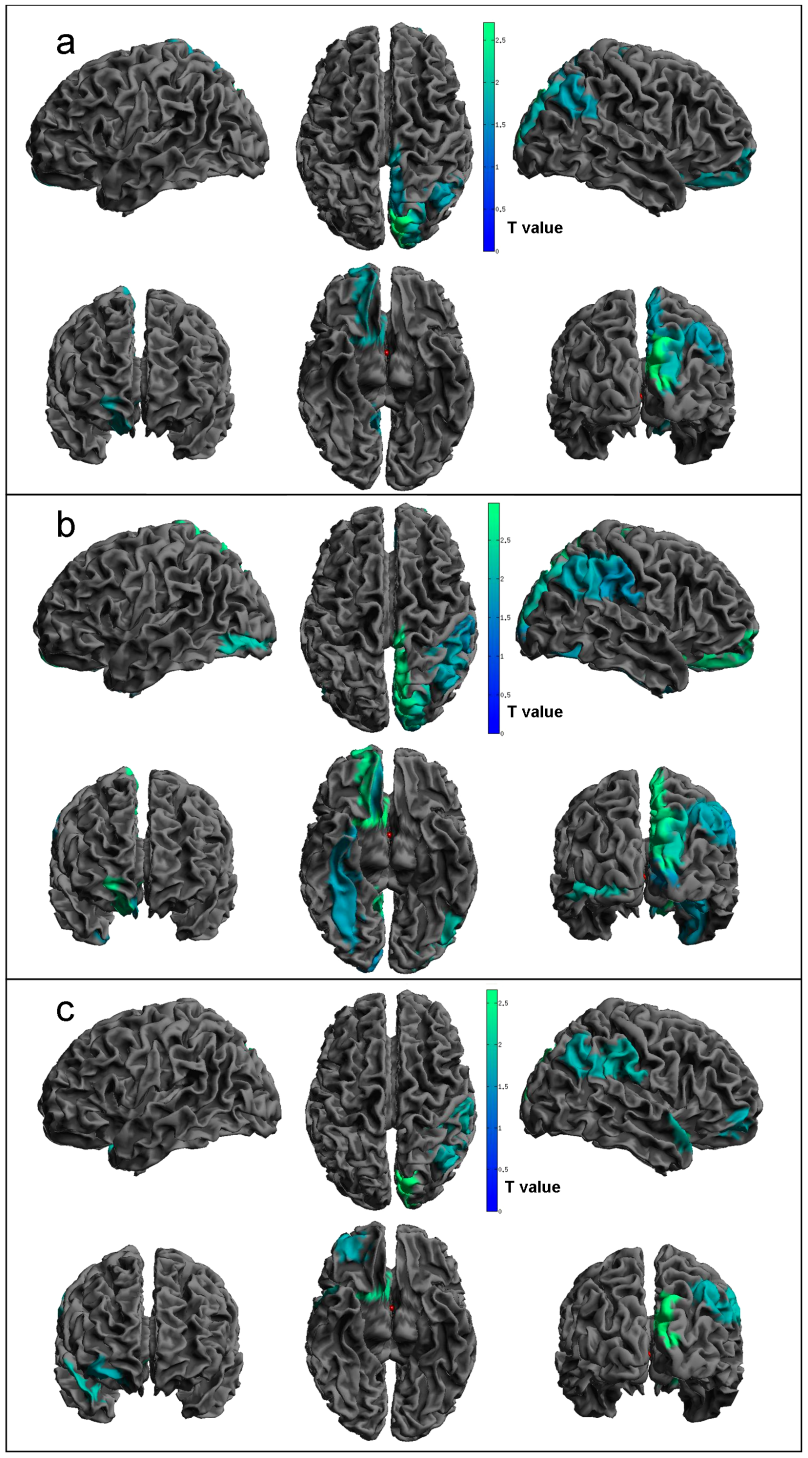
Regions of significant cortical thinning. Statistical threshold used T = 1.69, p<0.05. Panel a) PD vs. HC contrast; b) PD-CNT vs. HC; c) PD-MCI vs. HC.

**Figure 2:**
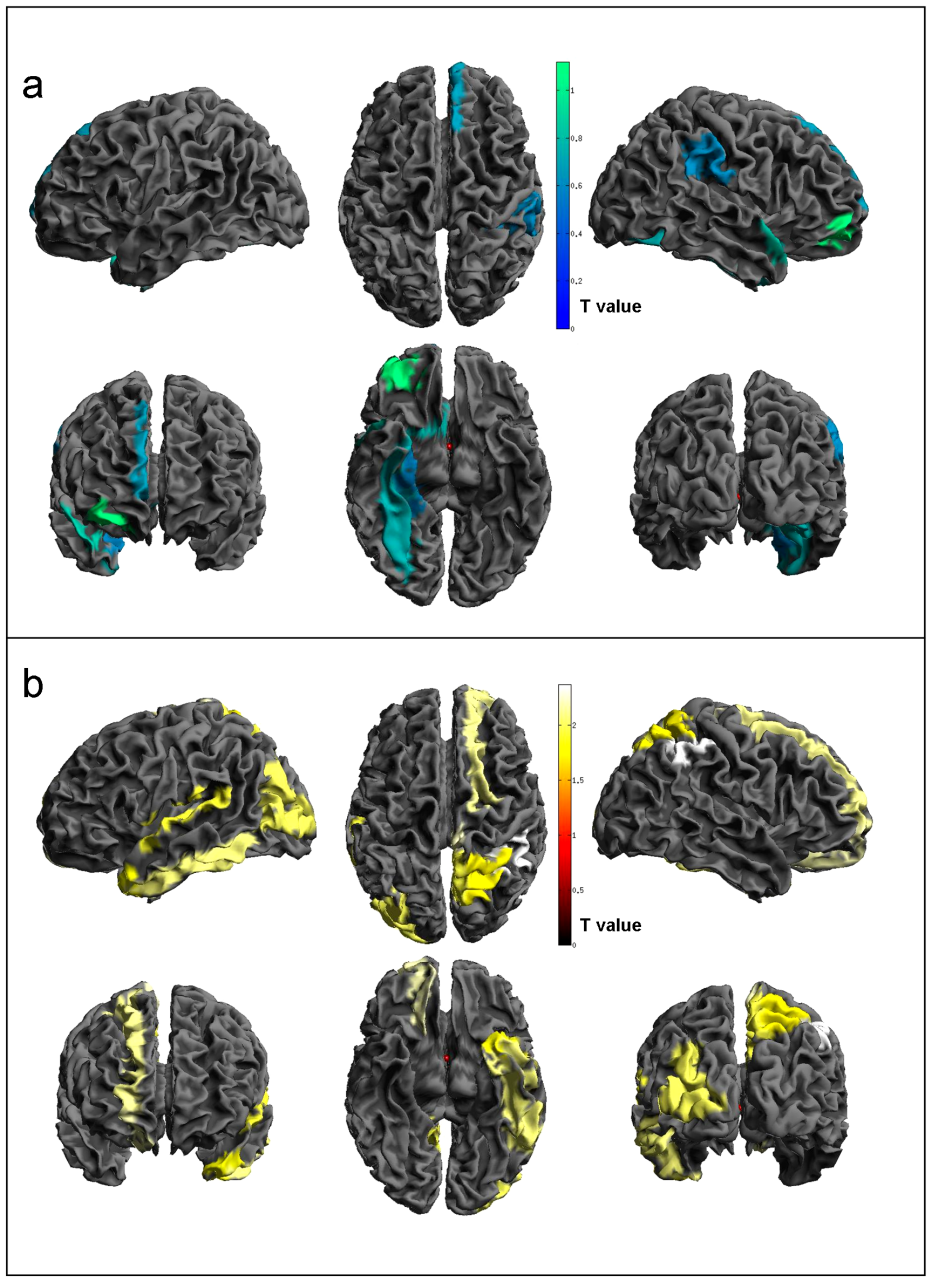
Regions of significant cortical thickness changes in PD-MCI vs. PD-CNT. Panel a) PD-MCI atrophy; b) PD-MCI hypertrophy. Statistical threshold used T = 1.69, p<0.05.

Details of the analysis are shown in Tables 2, 3 and 4 and Figures 1 and 2.

**Table 2:** Areas of cortical thinning in PD subgroups versus HC.

ATROPHY	AAL	T-value (Max)
**PD vs. HC**	Olfactory R	1,84
	Frontal Superior Orbital R	1,83
	Occipital Superior R	1,85
	Cuneus R	2,71*
	Angular R	1,71
	Precuneus R	1,7
**PD-CNT vs. HC**	Precuneus R	2,7*
	Cuneus R	2,58*
	Angular R	1,92
	Parietal Inferior R	1,97*
	Fusiform R	1,84
	Paracentral Lobule R	2.70*
	Supramarginal R	1,75
	Frontal Superior Orbital R	2,66*
	Rectus R	1,91
	Olfactory R	2,98*
	Occipital Superior R	2.18*
	Occipital Inferior L	2,16*
**PD-MCI vs. HC**	Cuneus R	2,67*
	Fusiform R	1,7
	Occipital Superior R	1,7
	Temporal Pole Superior R	1,89
	Frontal Midlle Orbital R	1,74
	Olfactory R	2,34*
	Supramarginal R	2,35
	Angular R	1,8

* = Cortical thickness comparison significant after AlphaSim threshold correction.

Remaining areas were not significant after correction and are reported as trend.

**Table 3:** Areas of cortical thickening in PD-MCI versus PD-CNT.

	AAL	T-value (Max)
**PD-MCI>PD-CNT**	Temporal Inferior L	2,09*
	Temporal Superior L	1,92
	Precuneus R	1,97*
	Paracentral Lobule R	2,24*
	Frontal Superior R	2,13*
	Frontal Superior Orbital R	2,21*
	Temporal Pole Middle L	1,92
	Occipital Middle L	2,01*
	Parietal Inferior R	2,37*
	Parietal Superior R	1,76

* = Cortical thickness comparison significant after AlphaSim threshold correction.

Remaining areas were not significant after correction and are reported as trend.

**Table 4:** Subcortical volume comparisons between PD and HC.

Regions	PD (Mean +/−Std mm^3^)	HC (Mean +/−Std mm^3^)	P-value
Left Hemisphere			
Putamen	4544,52±570,74	4862,1±477,14	0,031
Accumbens area	484,28±70,9	548,1±62,11	0,020
Right Hemisphere			
Global Cortical Thickness	2,28±0,17	2,39±0,18	0,009
Caudate	2971,52±229,59	3204,67±388,35	0,016
Putamen	4506,93±608,64	4932,38±608,41	0,020
Pallidum	1462,76±165,86	1582,14±236,34	0,055
Hippocampus	4083,31±458,11	4414,24±352,23	0,002*
Amygdala	1524,48±163,55	1742,38±174,45	0,000*
Accumbens area	467,62±72,48	538,1±73,96	0,003*

* = Comparisons significant after Bonferroni correction.

Remaining areas were not significant after correction and are reported as trend.

### Cognitive analysis

PD-MCI showed deficits in PM47 (p<0.01), TMT-A (p<0.05), phonological and semantic fluency task (p<0.5 and p<0.01 respectively), ROCF copy and immediate recall (p<0.001 and p<0.01), Stroop Color/Word Interference Test (time and error) (p<0.1 for both), FAB (p<0.01), Digit Span backward (p<0.02) and CDT (p<0.01). No differences were observed in BDI-II score between PD-CNT and PD-MCI. No PD patient reported hallucinations. Person's analysis showed significant correlations between PM47 and inferior temporal areas (p = 0.04), temporal pole (p = 0.04) and middle occipital areas (p = 0.04) in the left hemisphere. We also found that ROCF (copy and immediate recall) correlated with inferior temporal (p = 0.01), middle temporal (p = 0.03) and middle occipital areas (p = 0.02) in the left hemisphere, precuneus (p = 0.001) and superior orbito-frontal areas (p = 0.04) in the right hemisphere. Moreover a significant correlation was also observed between performance at the Stroop Color/Word Interference Test and precuneus (p = 0.01), paracentral lobe (p = 0.01), inferior parietal areas (p = 0.01) and superior frontal areas (p = 0.004) in the right and in the left middle-occipital areas (p = 0.02).

## Discussion

We found significant cortical thinning in the right occipital-parietal, in the middle frontal areas, in the right cuneus as well as volume reductions in the putamen bilaterally, in the left accumbens, in the right caudate and pallidum, in the right hippocampus and amigdale in PD patients compared with HC. These results underlie the presence of structural changes in PD patients at relatively early stage and support previous studies showing cortical thinning and atrophy in the neocortex and subcortical regions [36]–[49], [10], [12], [13,40,41]. However none of these studies investigated structural changes specifically in PD-MCI patients, and those comparing PD vs. HC have several limitations. In particular, one study used a larger sample than our (49 PD, 33 HC) but the authors did not perform an adequate cognitive battery [42]. Other two studies included a comprehensive neuropsychological evaluation but the whole PD sample was very small (15 PD and 15 HC) and neither MCI diagnosis [37] nor different MCI criteria [43] were applied [44]. Our study, so far, is the first to evaluate the cortico-morphometric pattern in a PD cohort including PD-MCI, diagnosed according with the most recent criteria [27], and investigating its association with cognitive profile.

Our PD-MCI patients showed significant regional thickening in right parietal-frontal areas and in left temporal-occipital areas relative to PD-CNT. Notably cortical thickening observed in PD-MCI vs. PD-CNT should be considered in the context of a general cortical thinning of PD when compared to HC.

Cortical thickness changes in PD-MCI may result from the action of complex mechanisms associated with plasticity and neuronal reorganization. Analysis of cognitive data in PD-MCI and their anatomical correlate may help understanding the nature of these mechanisms. PD-MCI showed specific cognitive impairments in attention-executive, visual-spatial and memory domains when compared with PD-CNT. These findings confirm the presence of heterogeneous cognitive abnormalities in PD-MCI and underlay the presence of posterior in addition to frontal cognitive deficits [2], [45], [5].

Correlation analysis revealed interesting structural-cognitive patterns of brain function and, more specifically, cortical thickening in areas involved in functional activities relevant for accomplishing tasks impaired in PD-MCI. Indeed, tasks such as PM47 involving visual-recognition and spatial assessment were associated with thickening of temporal areas and inferior occipital area. A functional MRI (fMRI) study found middle temporal lobe (MTL) activation during visual recognition task and additional occipital areas activation in non-PD subjects with MCI vs. HC. They explained these results as plastic brain reorganization in patients with medial temporal lobe damage [46]. Moreover visuo-spatial and visuo-perceptual (ROCF and Stroop Color/Word Interference Test) tasks correlated with left temporal inferior lobe and occipital middle areas as well as right precuneus and superior frontal areas thickening. There is evidence indicating that occipito-temporal and occipito-parietal pathways project to the prefrontal cortex and play a role in maintaining visuo-spatial information and object “on-line” [47]. fMRI studies showed that Stroop-like tasks activate fronto-parietal neural networks [48], [49]. Interestingly Kaufmann L. et al. [50] observed stronger and more extensive activation in individuals with MCI in fronto-parietal and occipital regions during Stroop Color/Word Interference Test reflecting more effortful information retrieval. Indeed, in our analysis we found anatomical correlates for a number of tests that exceeded those abnormal in PD-MCI. We believe that discrete brain areas are not univocally associated with a specific cognitive test but operate in the context of larger networks. This is in line with the concept that the same area can be functionally linked with different cognitive skills. Finally, fMRI data as well as our findings point to plasticity and reorganization of the non-motor system in PD-MCI, a condition that may herald dementia. We acknowledge that activation studies reflect changes in the extension of areas involved in specific tasks and not necessarily express variation in thickening. Although a strong correlation between hypometabolism and gray matter atrophy may be observed in presence of dementia [51], findings in non-PD MCI are inconsistent [52]–[55] and suggest a more complex relationship between grey matter atrophy and functional alterations. It is conceivable that to preserve cognitive skills, additional and/or compensatory brain areas “come into help” (which would result in relative hyperactivity or thickening) in cognitively demanding conditions trying to preserve a relative “cognitive stability”. These compensatory mechanisms may act only temporarily and herald, as disease progresses, a subsequent cognitive decline [56], [57]. Literature evidence shows paradoxical perfusion increases in patients at risk for AD (genetic risk, family history, MCI) and decreases in task related-fMRI activity in more cognitively impaired subjects [58], [59] suggesting a non-linear progression [60]. According to this non-linear hypothesis we would expect that cognitive severity is a modulating factor of neuronal responsivity. PD-MCI patients may initially exhibit hyper-activation or thickening in task-relevant brain structures reflecting compensatory mechanisms, while as cognitive abnormalities progress to dementia hypo-activation or thinning would develop.

We acknowledge that our data regard a relatively small number of patients and should be considered in the context of current MCI definition. The prevalence of PD-MCI is influenced by both the neuropsychological tools and cut-offs chosen to define cognitive impairment as clinically relevant [61], [62] as well as the definition of MCI itself, which is still debated [63]. We adopted the most recent criteria for PD-MCI based on statistical criteria (≤1.5 SD Z-score below the population's mean in at least two tests for the same cognitive domain or one test in at least two different domains) in an attempt to minimize the possibility of false negative but recognize that such criteria are not clinical and for this reason may affect sampling and results. However, it should be noted that there were areas that survived even after the multiple comparison correction, leading us to reject the idea of cortical thickness alteration in PD-MCI due to a type error I.

Although cortical thickness has been showed to have high sensitivity and specificity in detecting with more accuracy structural alteration change even at individual level [32], we believe this MRI methodology, is not sufficient to define the nature of cortical thickening found in our PD-MCI. Future studies using larger samples and including additional clinical and neuroimaging measurement (i.e. Diffusion Tensor Imaging) will be needed to further explore the complex relationship between cognitive phenotype, neuronal reorganization and structural degeneration and particularly in determining the evolution of this pattern of cortical abnormalities in PD-MCI.
